# Multicenter evaluation of two chemiluminescence and three lateral flow immunoassays for the diagnosis of COVID-19 and assessment of antibody dynamic responses to SARS-CoV-2 in Taiwan

**DOI:** 10.1080/22221751.2020.1825016

**Published:** 2020-10-01

**Authors:** Shey-Ying Chen, Yu-Lin Lee, Yi-Chun Lin, Nan-Yao Lee, Chia-Hung Liao, Yuan-Pin Hung, Min-Chi Lu, Jhong-Lin Wu, Wen-Pin Tseng, Chien-Hao Lin, Ming-Yi Chung, Chun-Min Kang, Ya-Fan Lee, Tai-Fen Lee, Chien-Yu Cheng, Cheng-Pin Chen, Chien-Hua Huang, Chun-Eng Liu, Shu-Hsing Cheng, Wen-Chien Ko, Po-Ren Hsueh, Shyr-Chyr Chen

**Affiliations:** a Department of Emergency Medicine, National Taiwan University Hospital, National Taiwan University College of Medicine, Taipei, Taiwan; b Center for Quality Management, National Taiwan University Hospital, College of Medicine, National Taiwan University, Taipei, Taiwan; c Department of Internal Medicine, Changhua Christian Hospital, Changhua, Taiwan; d Division of Infectious Diseases, Department of Internal Medicine, Taoyuan General Hospital, Ministry of Health and Welfare, Taoyuan, Taiwan; e Graduate Institute of Clinical Medicine, College of Medicine, Taipei Medical University, Taipei, Taiwan; f Department of Internal Medicine and Center for Infection Control, College of Medicine, National Cheng Kung University Hospital, Tainan, Taiwan; g Department of Medicine, College of Medicine, National Cheng Kung University, Tainan, Taiwan; h Division of Infectious Diseases, Department of Internal Medicine, Ministry of Health and Welfare Nantou Hospital, Nantou, Taiwan; i Department of Internal Medicine, Tainan Hospital, Ministry of Health & Welfare, Tainan, Taiwan; k Graduate Institute of Clinical Medicine, National Health Research Institutes, Tainan, Taiwan; l Division of Infectious Diseases, Department of Internal Medicine, China Medical University Hospital, Taichung, Taiwan; m Department of Microbiology and Immunology, School of Medicine, China Medical University, Taichung, Taiwan; n Department of Laboratory Medicine, National Taiwan University Hospital, National Taiwan University College of Medicine, Taipei, Taiwan; o Department of Pediatrics, National Taiwan University Hospital, Taipei, Taiwan; p School of Public Health, National Yang-Ming University, Taipei, Taiwan; q Institute of Clinical Medicine, National Yang-Ming University, Taipei, Taiwan; r School of Public Health, Taipei Medical University, Taipei, Taiwan; s Department of Internal Medicine, National Taiwan University Hospital, National Taiwan University College of Medicine, Taipei, Taiwan

**Keywords:** COVID-19, antibody response, lateral flow immunoassays, chemiluminescence immunoassays, cross-reactivity

## Abstract

This multicenter, retrospective study included 346 serum samples from 74 patients with coronavirus disease 2019 (COVID-19) and 194 serum samples from non-COVID-19 patients to evaluate the performance of five anti-severe acute respiratory syndrome coronavirus 2 (SARS-CoV-2) antibody tests, i.e. two chemiluminescence immunoassays (CLIAs): Roche Elecsys® Anti-SARS-CoV-2 Test (Roche Test) and Abbott SARS-CoV-2 IgG (Abbott Test), and three lateral flow immunoassays (LFIAs): Wondfo SARS-CoV-2 Antibody Test (Wondfo Test), ASK COVID-19 IgG/IgM Rapid Test (ASK Test), and Dynamiker 2019-nCoV IgG/IgM Rapid Test (Dynamiker Test). We found high diagnostic sensitivities (%, 95% confidence interval [CI]) for the Roche Test (97.4%, 93.4–99.0%), Abbott Test (94.0%, 89.1–96.8%), Wondfo Test (91.4%, 85.8–94.9%), ASK Test (97.4%, 93.4–99.0%), and Dynamiker Test (90.1%, 84.3–94.0%) after >21 days of symptom onset. Meanwhile, the diagnostic specificity was 99.0% (95% CI, 96.3–99.7%) for the Roche Test, 97.9% (95% CI, 94.8–99.2%) for the Abbott Test, and 100.0% (95% CI, 98.1–100.0%) for the three LFIAs. Cross-reactivity was observed in sera containing anti-cytomegalovirus (CMV) IgG/IgM antibodies and autoantibodies. No difference was observed in the time to seroconversion detection of the five serological tests. Specimens from patients with COVID-19 pneumonia demonstrated a shorter seroconversion time and higher chemiluminescent signal than those without pneumonia. Our data suggested that understanding the dynamic antibody response after COVID-19 infection and performance characteristics of different serological test are crucial for the appropriate interpretation of serological test result for the diagnosis and risk assessment of patient with COVID-19 infection.

## Introduction

Coronavirus disease (COVID-19), a potentially life-threatening respiratory tract disease, emerged at the end of 2019 in China and rapidly evolved to become a pandemic, resulting in severe repercussions to human health and life [1–3]. As of July 19, 2020, there have been 14,043,176 confirmed cases of COVID-19 and 597,583 (4.26%) deaths reported to the World Health Organization [4]. The early identification and isolation of patients with COVID-19 to prevent the transmission of the causative agent, severe acute respiratory syndrome coronavirus 2 (SARS-CoV-2), in the community are vital for curbing the pandemic. However, the identification of patients with COVID-19 is challenging because of its broad spectrum of clinical manifestations, ranging from asymptomatic infection to critical illness requiring intensive care [3,5]. The real-time reverse transcriptase-polymerase chain reaction (qRT-PCR) assay directly amplifies and detects SARS-CoV-2-specific viral nucleic acid sequences and has been an important and irreplaceable diagnostic tool for COVID-19 detection. However, the diagnostic sensitivity of qRT-PCR assay is influenced significantly by the timing of specimen collection during the disease course, site of specimen collection, and skill required during specimen collection [6,7]. It is therefore plausible that a significant proportion of patients with COVID-19 failed to be diagnosed using qRT-PCR [8,9].

Serological testing, i.e. detection of anti-SARS-CoV-2 antibodies simultaneously or separately, can be used as an adjuvant to qRT-PCR in the clinical diagnosis of acute COVID-19 [10–12]. Furthermore, serological testing can detect a previously undiagnosed SARS-CoV-2 infection among individuals for whom qRT-PCR was either falsely negative or not performed. Serological testing is therefore useful for studying the epidemiological seroprevalence of COVID-19 to obtain a more accurate estimate of the circulating dynamics and virulence of SARS-CoV-2 [13]. To date, many point-of-care or fully automated immunoassays for COVID-19 diagnosis have been developed and launched [11,12,14–17]. However, the performance and usefulness of different serological tests should be fully evaluated in clinical laboratories before its large-scale application into routine diagnostic protocols for patient management and pandemic control. The primary goal of this study was to evaluate the performance of five serological tests: Elecsys® Anti-SARS-CoV-2 (Roche Test), SARS-CoV-2 IgG (Abbott Test), Wondfo SARS-CoV-2 Antibody Test (Wondfo Test), ASK COVID-19 IgG/IgM Rapid Test (ASK Test), and Dynamiker 2019-nCoV IgG/IgM Rapid Test (Dynamiker Test) for the diagnosis of COVID-19. Further, previous studies have shown a correlation between the high-level upsurge of the anti-SARS-CoV-2 antibody response and tissue injury among patients with COVID-19 [18–20]. The secondary goal of this study was to validate the dynamic immune responses among patients with COVID-19 of different clinical severity with the individual or collective testing results of the five serological tests.

## Materials and methods

### Policy and status of the COVID-19 epidemic in Taiwan

In Taiwan, the respiratory tract specimens from patients who meet the reporting criteria for COVID-19 have to be submitted to virology laboratories validated and associated with the Centers for Diseases Control of Taiwan (Taiwan CDC) for SARS-CoV-2 qRT-PCR assay [21]. Three sets of primers and probes targeting the SARS-CoV-2 envelope (E), nucleocapsid (N), and RNA-dependent RNA polymerase (RdRp) genes were used. If the result of the first sample was negative for SARS-CoV-2, an additional SARS-CoV-2 qRT-PCR assay for another respiratory tract sample from the patient suggested of having COVID-19 was performed to minimize the risk of false-negative results using the qRT-PCR assay [22]. All qRT-PCR confirmed patients with COVID-19 have to be reported to the National Health Command Center and are mandatorily hospitalized in a negative-pressure isolation room to prevent the transmission of SARS-CoV-2 in the community. As of 19 July 2020, Taiwan has maintained a record of limited community transmission of COVID-19 and has 455 confirmed cases [23].

### Study design and patient enrollment

Six hospitals participated in this retrospective, observational study: National Taiwan University Hospital, National Cheng Kung University Hospital, Tao Yuan General Hospital, Ministry of Health and Welfare, Changhua Christian Hospital, Nantou Hospital, Ministry of Health and Welfare, and China Medical University Hospital. A total of 346 serum samples collected from 74 qRT-PCR confirmed patients with COVID-19 from the six participating hospitals were included for analysis. This study was approved by the institutional review board of all the participating hospitals, and the requirement for informed consent from each patient was waived (202003004RIND).

### Collection of serum samples and clinical data

We used the residual blood samples of patients with COVID-19 collected by the attending physicians providing regular medical care. All blood samples were collected on the date of venipuncture. If multiple blood samples were collected from a patient with COVID-19 on the same day, only the first sample collected that day was used for anti-SARS-CoV-2 antibody testing. The serum of the collected blood samples was stored at −20°C before testing.

We further included 194 control serum samples to evaluate the cross-reactivity and diagnostic specificity of the five antibody tests in this study: 70 from hospitalized patients with an acute respiratory infection (ARI) who tested negative ≥2 times using SARS-CoV-2 qRT-PCR and without any other confirmed aetiology for ARI collected in 2020, 50 from patients with ARI who tested negative ≥2 times using SARS-CoV-2 qRT-PCR and presence of specific microbiological aetiologies collected in 2020, 36 from patients who tested positive for any specific auto-antibody collected in 2020, and 38 from patients with pre-COVID-19 sera with presence of specific microbiological antigens or antibodies collected in 2019. Finally, control serum sample of positive result from any of the five serological tests was further examined if the presence of rheumatoid factor (RF) with Siemens N Latex RF Kit (Siemens BN™ II System) to examine the possibility of cross reaction between RF and anti-SARS-CoV-2 antibody [24,25].

Patient data including sex, age, comorbid medical condition, date of symptom onset, initial presentation, date of hospitalization, length of hospital stay, presence of pneumonia on chest roentgenogram, requirement of intensive care unit (ICU) admission, and survival status on hospital discharge were retrieved from electronic medical records of the participating hospitals.

### Chemiluminescence immunoassays (CLIA) for the detection of anti-SARS-CoV-2 antibodies

Elecsys® Anti-SARS-CoV-2 is an electrochemiluminescence immunoassay using the recombinant N protein for the detection of antibodies (including IgG) against SARS-CoV-2 with Cobas e immunoassay analyzers (Roche Diagnostics Basel, Switzerland) [15,26]. SARS-CoV-2 IgG, a chemiluminescent microparticle immunoassay, qualitatively detects IgG antibodies to the SARS-CoV-2 nucleocapsid protein (N protein) in human serum and plasma using the ARCHITECT i System (Abbott Laboratories, IL, USA) [14,27]. Test results were interpreted as positive if the electrochemiluminescent signal value of the Roche Test (cutoff index, COI) ≧1.0, or the chemiluminescent signal value of the Abbott Test (index [sample/calibrator], S/C) ≧1.4, as manufacturers’ instructions. The detailed information of the two assays is summarized in Table 1.

**Table 1.** Information on the two chemiluminescence immunoassays used for the diagnosis of coronavirus disease (COVID-19).

Parameter	Roche Elecsys® Anti-SARS-CoV-2	Abbott SARS-CoV-2 IgG
Company (city, country)	Roche Diagnostics (Basel, Switzerland)	Abbott Laboratories (IL, USA)
Targeting antibody	All antibodies (including IgG)	IgG
Immunoassay	Electrochemiluminescence	Chemiluminescent microparticle
Analyzer	Cobas e analyzers (e 411, e 601, and e 602)	ARCHITECT i System (i2000SR and i1000SR)
Qualitative analysis	Yes	Yes
Protein targeting	Nucleocapsid	Nucleocapsid
Specimen type(s)	Serum or plasma	Serum or plasma
Specimen amount required	20 µL (cobas e 411/cobas e 601/cobas e 602 modules) 10 µL (cobas e 601)	25 µL
Result interpretation	Cutoff index, COI <1.0: non-reactive (negative); ≥1.0: reactive (positive)	Index [Sample/Calibrator], S/C <1.4: negative; ≥1.4: positive
Testing time	18 min	15 min
Reported sensitivity or positive percent agreement (PPA) (95% CI) based on qRT-PCR results	204 serum samples from 69 symptomatic patients Days post PCR confirmation (sensitivity): 0–6 days (*n* = 116), 65.5% (56.1–74.1%) 7–13 days (*n* = 59), 88.1% (77.1–95.1%) ≥14 days (*n* = 29), 100% (88.1–100%)	122 serum samples from 31 patients Days post-symptom onset (PPA) <3 days (*n* = 4), 0.0% (0.0–60.2%) 3–7 days (*n* = 8), 25.0% (3.2–65.1%) 8–13 days (*n* = 22), 86.4% (65.1–97.1%) ≥14 days (*n* = 88), 100% (95.9–100.0%)
Reported specificity or negative percent agreement (NPA), (95% CI)	5272 samples (from routine diagnostic tests, blood donor tests, a common cold panel, and a coronavirus panel) obtained before December 2019 (10 false positives were detected). Specificity: 99.8% (99.7–99.9%)	997 specimens collected prior to September 2019 (4 false positives were detected) NAP: 99.6% (99.9–99.9%) 73 specimens collected in 2020 from patients exhibiting signs of respiratory illness who tested negative for SARS-CoV-2 by the qRT-PCR method NPA: 100% (95.1–100.0%)
Confirmed cross-reactivity with antibodies against non-coronaviruses	NA	One of five patients showed positivity for CMV IgG
Cross-reactivity with antibody against other coronaviruses SARS and MERS Other seasonal coronaviruses	NA Yes (40 potentially cross-reactive samples from individuals with past infection with coronaviruses HKU1, NL63, 229E, or OC43, confirmed by PCR)	NA NA
Registration	CE-IVD, USFDA	CE-IVD, US FDA
Reference	[24]	[25]

CE-IVD, Conformité Européenne *in vitro* diagnostic device; CMV, cytomegalovirus; MERS, Middle East respiratory syndrome; NA, not available; qRT-PCR, real-time reverse transcriptase-polymerase chain reaction; SARS, severe acute respiratory syndrome; S/C, sample/control; US FDA, Food and Drug Administration of the United States.

COVID-19, coronavirus disease 2019; ICU, intensive care unit; ECMO, extracorporeal membrane oxygenation.

### Lateral flow immunoassays (LFIA) for the detection of anti-SARS-CoV-2 antibodies

Three qualitative lateral flow immunoassays (LFIA) for detecting anti-SARS-CoV-2 antibodies were evaluated in this study: Wondfo SARS-CoV-2 Antibody Test (Guangzhou Wondfo Biotech Co., Ltd., China), ASK COVID-19 IgG/IgM Rapid Test (TONYAR Biotech Inc. Taiwan), and Dynamiker 2019-nCoV IgG/IgM Rapid Test (Dynamiker Biotechnology [Tianjin] Co., Ltd., China). All the three rapid tests detected either anti-SARS-CoV-2 IgG and IgM antibodies separately (ASK Test and Dynamiker Test) or total antibody (Wondfo Test) within 5–15 min. The viral protein labelled was the SARS-CoV-2 Spike protein (S protein) in the Wondfo and ASK Tests and N protein in the Dynamiker Test. These tests used either the whole blood, serum, or plasma as the testing specimen and required only 10–20 µL of sample volume. Positive results were interpreted as the presence of control line and either IgG or IgM test line for ASK Test and Dynamiker Test, or control line and total antibody test line in Wondfo Test. A weakly positive result (any shade of colour in the test lines) of an antibody rapid testing was considered positive according to the manufacturers’ instructions [18,28–30].

### Definitions

Test-specific seroconversion in a patient with COVID-19 was defined as the earliest date on which a positive anti-SARS-CoV-2 antibody response was detected in a serum sample using a specific antibody test. The presumptive seroconversion was an ideal approximation of the true biological seroconversion. It was defined as the earliest date on which a positive anti-SARS-CoV-2 antibody response was detected in a serum sample using any one of antibody tests in this study. COVID-19-compatible presentation included fever (≥38°C), respiratory tract, constitutional, anosmic, dysgeusic, and diarrheal symptom. Date of symptom onset was defined as the onset date of the first aforementioned COVID-19-compatible symptom reported by a symptomatic COVID-19 patient. Time to seroconversion detection was defined as the duration from the date of symptom onset to the date of seroconversion. The diagnostic sensitivity of an antibody test was defined as the percentage of serum samples from confirmed patients with COVID-19 that is positive for antibodies against SARS-CoV-2, whereas the diagnostic specificity of an antibody test was defined as the percentage of serum samples from control patients that is negative for antibodies against SARS-CoV-2.

### Statistical analysis

We calculated means and standard deviations (SDs) for age variables and percentages for the categorical variables. The measurement agreements between different antibody tests were evaluated with Cohen's kappa (к) statistics. The cumulative probabilities of detection of seroconversion for a specific antibody test were obtained using the Kaplan–Meier method. The difference in the cumulative probability of detection of seroconversion between the five antibody tests was evaluated using the log rank test. Further, the difference in cumulative probability of detection of seroconversion between patients with COVID-19 with and without pneumonia was also investigated using the log rank test. Data were analysed using SPSS for Windows (IBM SPSS Statistics v26). All *p*-values are two-sided, and a *p*-value less than 0.05 was considered statistically significant.

## Results

### Clinical characteristics of patients with COVID-19

A total of 346 serum samples were collected from 74 consecutively qRT-PCR-confirmed COVID-19 patients who were treated at six participating hospitals between 23 January 2020 and 31 May 2020, were enrolled in this study. The mean patient age was 38.5 years (SD, 16.2 years). Forty-one (55.4%) patients were men and 67 (90.5%) of them had no significant comorbid or surgical condition. All the 74 enrolled COVID-19 patients reported at least one COVID-19-compatible symptom. Lower respiratory tract symptoms were the predominant symptom at the time of diagnosis (66.2%), followed by upper airway symptoms (62.2%), and fever (45.9%). Twenty-eight (37.8%) patients developed pneumonia during hospitalization, among whom five (6.8%) required ventilator support and intensive care (Table 2).

**Table 2.** Clinical characteristics of the 74 patients with confirmed COVID-19, stratified based on the availability of sequential samples for estimating the date of seroconversion.

Characteristics^a^	All patients	Seroconversion assessable^b^	Seroconversion not assessable^b^	*p*
No. of patients/no. of serum samples	74/346	48/230	26/116	
Age (years)	38.5 ± 16.2	37.8 ± 16.3	39.9 ± 16.2	0.600
Male sex	41 (55.4)	26 (54.2)	15 (57.7)	0.771
*Comorbid medical condition*				
Diabetes mellitus	3 (4.1)	1(2.1)	2 (7.7)	0.281
Malignancy	2 (2.7)	0 (0)	2 (7.7)	0.120
Coronary artery disease	2 (2.7)	2 (4.2)	0 (0)	0.538
Congestive heart failure	1 (1.4)	1 (2.1)	0 (0)	1.000
*Initial presentation*				
Fever	34 (45.9)	22 (45.8)	12 (46.2)	0.979
Headache	18 (24.3)	13 (27.1)	5 (19.2)	0.452
Myalgia	12 (16.2)	6 (12.5)	6 (23.1)	0.323
Malaise	19 (25.7)	11 (22.9)	8 (30.8)	0.460
Upper airway symptoms^c^	46 (62.2)	29 (60.4)	17 (65.4)	0.674
Low respiratory tract symptoms^d^	49 (66.2)	32 (66.7)	17 (65.4)	0.911
Diarrhea	21 (28.4)	14 (29.2)	7 (26.9)	0.838
*Treatment outcome*				
Length of hospital stay (days)	28.5 ± 13.4	28.2 ± 12.6	29.0 ± 15.0	0.815
Diagnosis of pneumonia	28 (37.8)	20 (41.7)	8 (30.8)	0.356
ICU admission	5 (6.8)	2 (4.2)	3 (11.5)	0.337
Ventilator required	5 (6.8)	2 (4.2)	3 (11.5)	0.337
ECMO support received	1 (1.4)	0 (0)	1 (3.8)	0.351
Hospital mortality	1 (1.4)	0 (0)	1 (3.8)	0.351
*Sample collection*				
Available sample number	4.7 ± 4.6	4.8 ± 2.7	4.5 ± 7.1	0.773
No. of days between symptom onset and sample collection	11.4 ± 14.8	5.7 ± 4.0	22.0 ± 20.8	<0.001

^a^ All values are expressed as mean ± SD or number (percentage).

^b^ Patients for whom sequential samples adequate for judging the approximate date of seroconversion were available.

^c^ Includes rhinorrhea, nasal stiffness, sore throat, and hoarseness.

^d^ Includes cough, productive sputum, dyspnea, and chest pain.

Of the 74 patients with COVID-19, 48 patients had sequential serum samples adequate for estimating the date of seroconversion. Clinical and serological data of these 48 patients were used to evaluate the utility of different antibody tests in the early detection of seroconversion after COVID-19 infection. The clinical characteristics of these 48 patients and the other 26 patients who were excluded from seroconversion analysis are compared in Table 2. The date of first serum sample collection from symptom onset was significantly different between these two patient groups (5.7 ± 4.1 vs. 22.0 ± 20.8, *p* < 0.001).

### Anti-SARS-CoV-2 antibody response among patients with COVID-19

A total of 346 serum samples were obtained from the 74 patients with COVID-19 (the number of samples obtained from each individual patient ranged from 1 to 38 samples; median, 4 samples) at different time points during the disease course (the duration from symptom onset to the sampling date ranged from 1 to 93 days; median, 7 days). Of the 346 serum samples, 270 (78.0%) showed anti-SARS-CoV-2 antibodies on using the Roche Test, 259 (74.9%) on using the Abbot Test, 267 (77.2%) on using the Wondfo Test, 275 (79.5%) on using the ASK Test, and 244 (70.5%) on using the Dynamiker Test. On using the ASK and Dynamiker Tests, which are antibody tests that detect IgM and IgG antibody separately, IgM antibody was detected in 222 (64.2%) and 239 (69.1%) samples and IgG antibody was detected in 222 (64.2%) and 237 (68.5%) samples, respectively.

**Figure 1.**
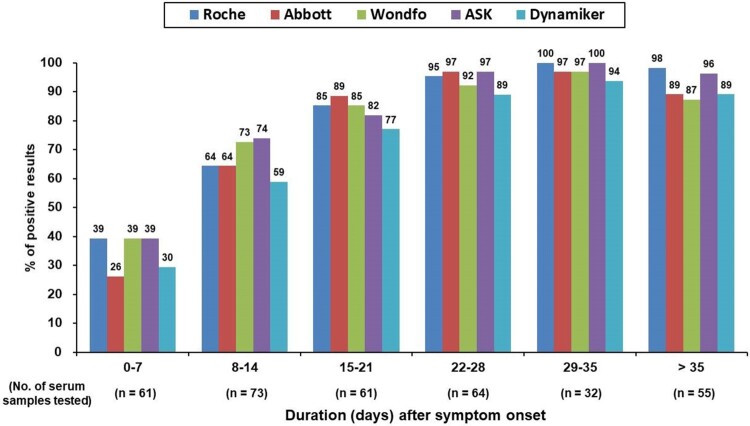
Percentage of samples showing positive antibody findings when examined using the five studied serological tests after symptom onset.

**Figure 2.**
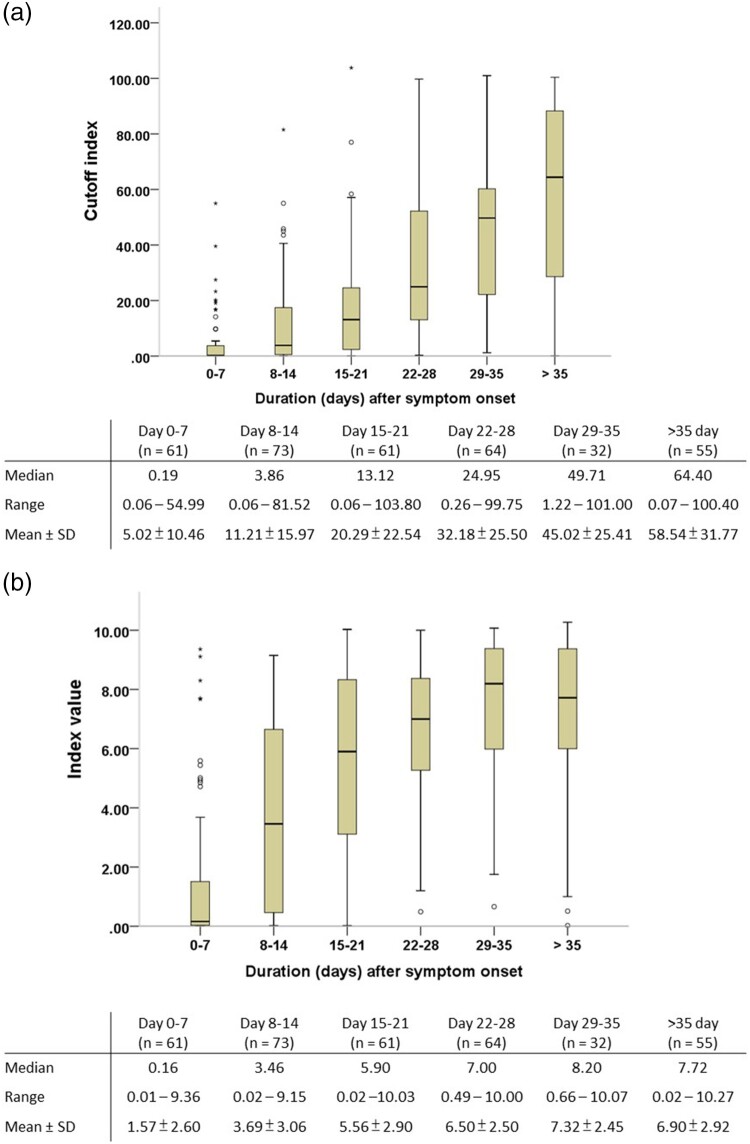
Chemiluminescent signal values of the two chemiluminescence immunoassays for anti-SARS-CoV-2 antibodies detection after symptom onset. (A) Roche Elecsys® Anti-SARS-CoV-2 Assay. (B) Abbott SARS-CoV-2 IgG Assay.

The antibody responses at different time intervals after symptom onset were further evaluated using the five antibody tests (Figure 1). The electrochemiluminescent signal value of the Roche Test (cutoff index, COI) and the chemiluminescent signal value of the Abbott Test (index [sample/calibrator], S/C) at different time intervals after symptom onset were shown in Figure 2(A and B), respectively. Percentage of positive testing result of all the five serological tests and chemiluminescent signal values of the two CLIA serological tests for anti-SARS-CoV-2 antibodies increased as the duration after symptom onset. One 29-year-old woman had negative test results at days 8, 11, 14, 19, and 74 of symptom onset, with all five serological tests. Including serological testing data from this woman, all tests had high diagnostic sensitivity of more than 90% after 21 days of symptom onset. Between-test measurement agreements among the different anti-SARS-CoV-2 antibody tests were further evaluated and are detailed in Table 3. The Wondfo Test had a higher measurement agreement with the ASK Test (к value = 0.830) than with the other three antibody tests in this study. In contrast, the Roche, Abbott, and Dynamiker Tests had a higher measurement agreement (к value ranged from 0.745 to 0.789) than with the Wondfo or ASK Test (к value < 0.683). For the two antibody tests that detected IgM and IgG antibodies separately, there was high measurement agreement for IgM and IgG detection within the Dynamiker Test (к value = 0.919) but not within the ASK Test (к value = 0.334).

**Table 3.** Agreement among findings from the five anti-SARS-CoV-2 antibody tests.

		Roche^a^	Abbott^b^	Wondfo^c^	ASK^d^			Dynamiker^e^		
					IgM	IgG	IgG/IgM	IgM	IgG	IgG/IgM
Roche^a^		–	0.784	0.659	0.409	0.533	0.681	0.728	0.716	0.745
Abbott^b^		–	–	0.683	0.428	0.617	0.657	0.757	0.759	0.789
Wondfo^c^		–	–	–	0.570	0.611	0.830	0.621	0.609	0.635
ASK^d^	IgM	–	–	–	–	0.334	0.632	0.436	0.400	0.411
	IgG	–	–	–	–	–	0.632	0.605	0.671	0.621
	IgG/IgM	–	–	–	–	–	–	0.627	0.630	0.642
Dynamiker^e^	IgM	–	–	–	–	–	–	–	0.919	0.966
	IgG	–	–	–	–	–	–		–	0.952
	IgG/IgM	–	–	–	–	–	–			–

^a^ Roche Elecsys® Anti-SARS-CoV-2 assay.

^b^ Abbott SARS-CoV-2 IgG assay.

^c^ Wondfo SARS-CoV-2 Antibody Test.

^d^ ASK COVID-19 IgG/IgM Rapid Test.

^e^ Dynamiker 2019-nCoV IgG/IgM Rapid Test.

### Cross-reactivity and diagnostic specificity of the anti-SARS-CoV-2 antibody tests

The results of cross-reactivity analysis of the 194 control serum samples from the patients without COVID-19 with the five antibody tests are detailed in Table 4. Two samples showed reactivity with the Roche Test; both were anti-cytomegalovirus (CMV) antibody-positive serum sample. Four samples showed reactivity with the Abbott Test. Among them 3 serum samples showed anti-CMV IgM/IgG antibodies, and 1 serum sample showed autoantibodies. None of the above six serum samples showing reactivity with either Roche Test or Abbott Test was positive for RF (all < 10.1 IU/mL). No control serum sample showed positive results with the 3 serological tests with lateral flow immunoassays. After 21 days of symptom onset, the Roche, Abbott, Wondfo, ASK, and Dynamiker Tests had diagnostic sensitivities (% [95% Confidence Interval {CI}]) of 97.4% (93.4–99.0%), 94.0% (89.1–96.8%), 91.4% (85.8–94.9%), 97.4% (93.4–99.0%), and 90.1% (84.3–94.0%), respectively, and diagnostic specificities of 99.0% (96.3–99.7%), 97.9% (94.8–99.2%), 100.0% (98.1–100.0%), 100.0% (98.1–100.0%), and 100.0% (98.1–100.0%), respectively (Supplement Table S1)**.**

**Table 4.** Evaluation of cross-reactivity for the five anti-SARS-CoV-2 antibody tests.

Cohort	No. of specimens tested	No. of specimens with positive results				
		Roche Elecsys® Anti-SARS-CoV-2 assay	Abbott SARS-CoV-2 IgG assay	Wondfo SARS-CoV-2 antibody test	ASK COVID-19 IgG/IgM rapid test	Dynamiker 2019-nCoV IgG/IgM rapid test
Total no. of specimens tested	194	2	4	0	0	0
*For patients treated between January 31 and May 31, 2020*						
Patients with ARI and negative qRT-PCR results on ≥2 tests, without other confirmed etiologies for ARI	70	0	0	0	0	0
Patients with ARI and negative qRT-PCR results on ≥2 tests showing the presence of specific etiologies (respiratory tract [antigens] or serum [antibodies])	50	2	2	0	0	0
Coronavirus OC43	2	0	0	0	0	0
Coronavirus 229E	1	0	0	0	0	
CMV IgG/IgM	7	1 (1.56)^a^	0	0	0	0
CMV IgG/IgM and HSV-IgM	1	0	0	0	0	0
CMV IgG	11	1 (1.42)^a^	1 (1.98)^a^	0	0	0
CMV IgM and HSV-IgM	1	0	0	0	0	0
CMV IgM and HSV-IgM and EBV VCA-IgM	1	0	1 (1.61)^a^	0	0	0
HSV-IgM	1	0	0	0	0	0
EBV VCA-IgM	5	0	0	0	0	0
*Mycoplasma pneumoniae* IgM	5	0	0	0	0	0
*Chlamydophila trachomatis* IgG	5	0	0	0	0	0
Respiratory syncytial virus (positive antigen test)	2	0	0	0	0	0
Influenza A virus (positive rapid antigen test)	4	0	0	0	0	0
Influenza B virus (positive rapid antigen test)	4	0	0	0	0	0
Patients showing the presence of any specific auto-antibodies^b^ in their sera (May 1 to May 31, 2020)	36	0	1 (2.17)^a,c^	0	0	0
*For patients treated between August 1 and December 31, 2019*						
Patients showing the presence of specific antigens/antibodies in their sera	38	0	1	0	0	0
*Mycoplasma pneumoniae* IgM	15	0	1 (4.54)^a,d^	0	0	0
*Chlamydophila pneumophilia* IgM	5	0	0	0	0	0
EBV VCA-IgA	10	0	0	0	0	0
Respiratory syncytial virus (positive antigen test)	1	0	0	0	0	0
Influenza A virus (positive rapid antigen test)	3	0	0	0	0	0
Influenza B virus (positive rapid antigen test)	4	0	0	0	0	0

SARS-CoV-2, severe acute respiratory tract syndrome coronavirus 2; COVID-19, coronavirus disease 2019; ARI, acute respiratory tract infection; qRT-PCR, real-time reverse transcriptase-polymerase chain reaction; CMV, cytomegalovirus; HSV, herpes simplex virus; EBV, Epstein-Barr virus; VCA, viral capsid antigen.

^a^ The serum sample was additionally tested for the presence of rheumatoid factor, which showed a negative result (<10.1 IU/mL).

^b^ Includes anti-nuclear, anti-ENA, anti-SS-A, anti-RNP, anti-SCL-70, anti-CCP, anti-Jo-1, anti-B2 GP1, anti-cardiolipin IgM, anti-cardiolipin IgG, anti-CENP, anti-MPO (*P*-ANCA), and anti-ribosomal-*P* antibodies, anti-basement membrane zone antibodies, anti-intercellular substance antibodies, anti-mitochondrial antibodies, and anti-gastric parietal cell antibodies.

^c^ Serum was positive for anti-nuclear (1:1280 + homogeneous), anti-ribosomal-*P* (241.08), and anti-ds DNA (237.79 WHO units/mL) antibodies but negative for anti-CMV IgM and IgG.

^d^ Also positive for anti-CMV IgG antibodies (no anti-CMV IgM antibodies).

### Assessment of antibody dynamic responses to SARS-CoV-2 infection severity

**Figure 3.**
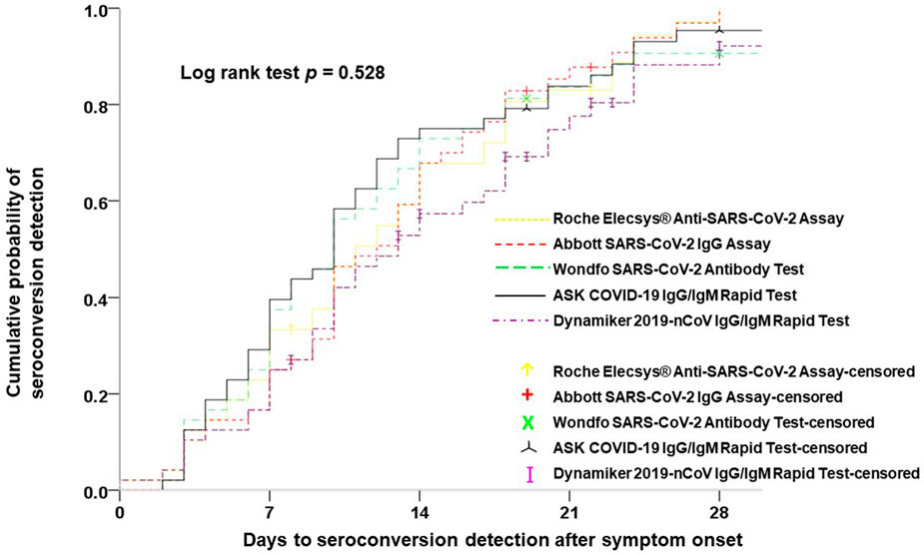
Parallel comparisons of the cumulative probability of seroconversion detection among the five studied serological tests.

Of clinical and antibody test data from all enrolled patients with COVID-19, data from 48 patients who had sequential serum samples adequate for estimating the date of seroconversion were used to evaluate the ability of early detection of seroconversion among the different anti-SARS-CoV-2 antibody tests and to assess the antibody dynamic response after SARS-CoV-2 infection. Analysis of the ability of early diagnosis of COVID-19 among the five antibody tests was carried out using the Kaplan–Meier test for detection of the cumulative probability of seroconversion (Figure 3). There was no statistical difference in the results after 7 (*p* = 0.416), 14 (*p* = 0.297), 21 (*p* = 0.554), and 28 days (*p* = 0.528) of symptom onset on the basis of the log rank test.

**Figure 4.**
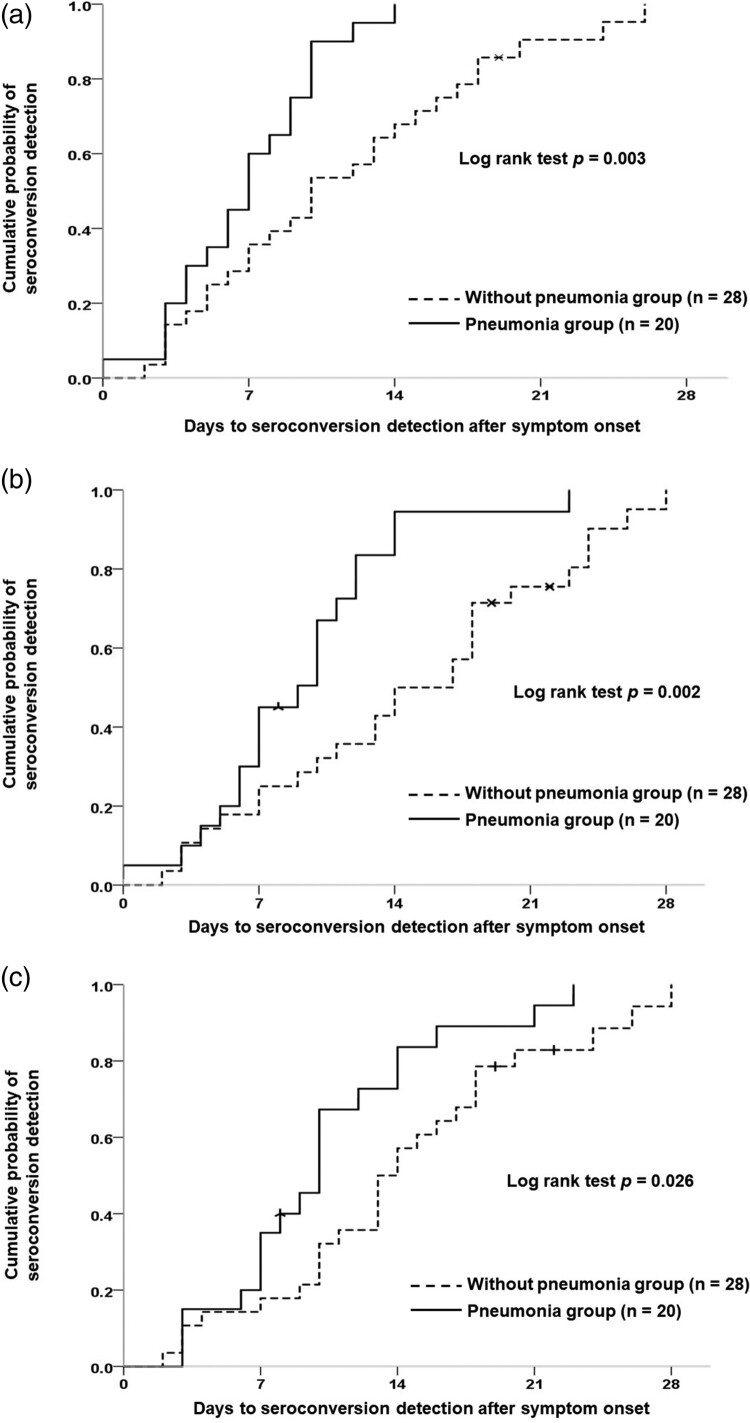
Detection of seroconversion in patients with COVID-19 with or without pneumonia. (A) Presumptive seroconversion based on earliest detection by any serological test. (B) Roche Elecsys® Anti-SARS-CoV-2 Assay. (C) Abbott SARS-CoV-2 IgG Assay.

**Figure 5.**
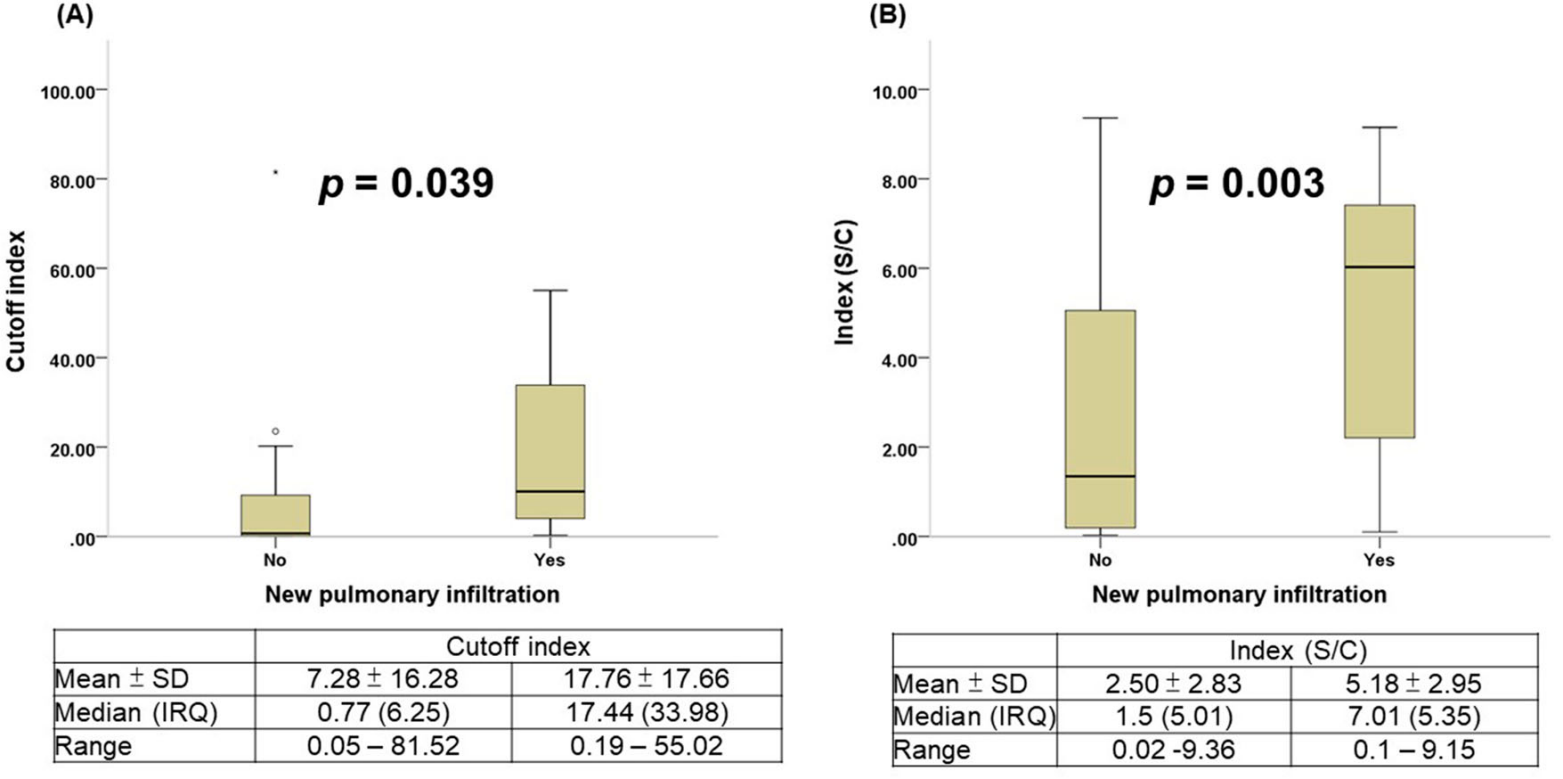
Comparison of chemiluminescent signal values in patients with COVID-19 with or without pneumonia. (A) Roche Elecsys® Anti-SARS-CoV-2 Assay. (B) Abbott SARS-CoV-2 IgG Assay.

Twenty of the 48 patients with COVID-19 developed radiographic evidence of pneumonia. Comparison of the time to presumptive seroconversion between patients with COVID-19 with and without pneumonia is shown in Figure 4(A). COVID-19 infected patients with pneumonia showed a shorter seroconversion time than did those without pneumonia (log rank test *p* = 0.003). Similar results were observed with test-specific seroconversion in the two CLIA anti-SARS-CoV-2 antibody tests (Figure 4(B and C)) and the three LFIA anti-SARS-CoV-2 antibody tests (Supplement Figure S1). Finally, patients with COVID-19 with and without pneumonia showed significant differences in the electrochemiluminescent signal of the Roche Test (COI) (17.76 ± 17.66 vs. 7.28 ± 16.28, *p* = 0.039) and the chemiluminescent signal of the Abbott Test (S/C) (5.18 ± 2.95 vs. 2.50 ± 2.83, *p* = 0.003) (Figure 5).

## Discussion

This study parallelly evaluated the performance of five anti-SARS-CoV-2 antibody tests in the diagnosis and severity assessment of COVID-19. There are four major findings of this study. First, the performance of serological tests in the diagnosis of COVID-19 highly depends on the time during the disease course and it stabilized 21 days after symptom onset. Second, although there was no significant difference among the five serological tests in the early detection of seroconversion after COVID-19 development, variations in the diagnostic sensitivity and between-test measurement agreements existed among the five studies serological tests. Third, cross-reactivity with anti-CMV and autoimmune antibodies was observed in anti-SARS-CoV-2 antibody tests using the chemiluminescence method but not in serological tests using LFIA method. Forth, patients with COVID-19 with pneumonia exhibited an earlier anti-SARS-CoV-2 antibody response and a higher post-symptom onset week-2 antibody chemiluminescent signal than did patients with COVID-19 without pneumonia. These findings are important for the appropriate application and interpretation of the results of serological tests by first-line physicians for diagnostic and therapeutic decision making during the COVID-19 pandemic.

Many automated or point-of-care serological tests have been rapidly manufactured to meet the urgent clinical and epidemiological needs to cope with the unprecedented spread and tremendous impact of the COVID-19 pandemic [14,15,17]. An ideal serological test for SARS-CoV-2 should have a high diagnostic sensitivity, low or no cross-reactivity with other existing antibodies, and a high sample throughput to prevent the delay of timely therapeutic decisions due to false-negative results, erroneous assumption of immunity to SARS-CoV-2 due to false-positive result, and facility collapse due to staff burn-out [31]. Previous studies have reported a high performance of various serological tests after the plateau phase of antibody formation, mostly 3 weeks after symptom onset [11,14,15,18]. Our study, however, found diverse diagnostic sensitivities for the different serological test. The failure of a serological test to detect anti-SARS-CoV-2 antibodies might be the result of the high detection limit of the test, low antibody titre, or a delay in or absence of immune response to SARS-CoV-2 after infection. For example, in this study, one 29-year-old woman with qRT-PCR-confirmed COVID-19 showed multiple negative results for sequential serum samples with all five serological tests till 74 days after symptom onset. Because this woman had no known immunosuppressive disease and was not under immunosuppressive medication, transient SARS-CoV-2 existence without induction of a measurable antibody response is a plausible explanation for the negative test results. Similar observations have been reported in the recent literature [32]. Therefore, our study demonstrates the effect of individual variations in immunological responses to infection on the performance of serological tests.

Another interesting observation was the measurement agreement grouping phenomenon of the five studied serological tests. In this study, the Roche, Abbott, and Dynamiker Tests had a high between-measurement agreement with each other but not with the Wondfo and ASK Tests. All of these three tests detect nucleocapsid protein-specific antibodies. In contrast, both the Wondfo and ASK Tests detect antibodies targeting the SARS-CoV-2 spike protein and showed high between-measurement agreements among each other but not with the other three tests in this study. Although there was no significant difference in the timing of seroconversion detection, positive percentages of the 346 serum samples were relatively higher in the Wondfo and ASK Tests (77.2% ∼ 79.5%) than in the other three tests (70.5% ∼ 78.0%). This suggests a plausible methodology-specific effect on the diagnostic sensitivity of a serological test.

Cross-reaction with autoantibodies or antibodies responding to other microorganisms, resulting a false-positive result in a serological test is important and should be comprehensively evaluated for accurate interpretation of test results. Previous studies have reported cross-reactivity with sera containing antibodies positive for CMV, Epstein–Barr virus (EBV), and systemic lupus erythematous in the Roche Test [15,16]. Our study further identified that cross-reactivity occurred not only in the Roche Test but also in the Abbott Test. Of the 6 serum samples from patients without COVID-19 that yielded positive results on the Roche Test or Abbott Test, 3 were anti-CMV IgM/IgG antibody-positive and 1 was autoantibody-positive. The other 2 serum samples, although containing different antibodies, also were positive for anti-CMV antibodies. Cross-reactivity, however, was not observed in serological tests using LFIAs. This is an interesting observation deserved further exploration. Difference in labelling viral protein between serological tests might explain the absence of cross reaction with anti-CMV antibody in Wondfo Test and ASK Test, which use S protein as labelling viral protein. The Dynamiker Test, similar to the Roche Test and Abbott Test, uses N protein as labelling viral protein but also showed no cross reaction with anti-CMV antibody. Because for anti-CMV containing sera with false-positive result, the COI (Roche Test) or S/C (Abbott Test) values were generally low. We therefore suppose that chemiluminescence immunoassay method combining analyzer might be more sensitive than visual lateral flow immunoassay method in the detection of weak cross-reactivity between anti-SARS-CoV-2 and anti-CMV antibody.

On the basis of these observations, positive results of serological tests should be carefully interpreted, especially in communities with a low prevalence and low local transmission rates of COVID-19, like Taiwan [23], and with a high prevalence of CMV infection, as well as in patients with known or possible autoimmune disease [33–35]. From the above findings, it can be concluded that although different tests show comparable results in detecting anti-SARS-CoV-2 antibodies, factors such as test-specific methodology, cutoff value determination, biological variation among individual patients, and the presence of cross-reactive antibodies remain important and effect the overall performance of serological tests.

As of 19 July 2020, a total of 79,645 people in Taiwan had been tested for SARS-CoV-2 and 455 (0.57%) were confirmed as having COVID-19, with an incidence rate of 19.3 per 1,000,000 people. Among the 455 COVID-19 patients, only 55 (12.1%) had contracted the disease from local transmission. To date, Taiwan has gone >100 days without reporting a single case of local transmission of COVID-19 [23]. Therefore, if a CLIA method (either Roche Test or Abbott Test) was applied for seroprevalence study for mass surveillance or among different risk populations in countries with a low prevalence of COVID-19 like Taiwan, further tests, including LFIAs, evaluation of the presence of anti-CMV antibodies or autoantibodies, the western blotting method, and even qRT-PCR assays for respiratory secretions of enrolled participants with positive results on CLIAs, are needed to confirm or exclude the presence of SARS-CoV-2 RNA or anti-SARS-CoV-2 antibodies [32,36,37].

In our previous study using LFIAs for detecting anti-SARS-CoV-2 antibodies, we demonstrated that patients with COVID-19 with pneumonia exhibited earlier seroconversion than did patients with COVID-19 without pneumonia [18]. In this study, we consolidated this observation from serological tests of LFIA method to tests of CLIA method with a large case number. In addition, we further demonstrated a significant antibody chemiluminescent signal difference between patients with COVID-19 with and without pneumonia. Although designed for qualitative detection of antibody to SARS-CoV-2, our studies found that information on signal number from CLIAs during the second week of symptom onset might be useful for clinicians to alter the risk of subsequent progression at an early phase of COVID-19.

This study has some limitations. First, it was a multicenter study. Though we used a standardized patient reporting form for clinical data collection, investigators from different participating hospitals were involved during data collection, which could have contributed to information bias. Second, as this was a non-protocolized retrospective study, serum samples tested at different post-symptom stages were not standardized; therefore, this study is also subject to information bias due to laboratory data. Third, owing to a small number of cases, significant differences in diagnostic performance between different serological tests may not have been observed. Fourth, concomitant infection with other respiratory tract viral pathogens, especially other coronaviruses, was inadequately evaluated among patients with COVID-19 in this retrospective study. Finally, because plain chest roentgenography is less sensitive than computed tomography in detecting parenchymal changes of viral pneumonia, we could not exclude the possibility that COVID-19 pneumonia patients with subclinical pulmonary infiltration were misclassified as non-pneumonia patients. Due to the above limitations, the validity of our study findings needs to be further confirmed in a prospectively designed diagnostic accuracy study.

In conclusion, in addition to qRT-PCR, serological testing may be a useful tool for the diagnosis of patients with active or past COVID-19. However, the diagnostic sensitivity of serological tests for COVID-19 is highly dependent on antibody dynamics, which reach a stable state after 3 weeks of symptom onset. Furthermore, cross-reaction against anti-CMV antibodies and autoantibody-containing sera was observed when using chemiluminescence immunoassay-based tests, although this was not observed with LFIA. Patients with COVID-19 complicated by pneumonia exhibited earlier anti-SARS-CoV-2 antibody response and a higher antibody chemiluminescent signal than those without pneumonia. Our study findings are informative and provide supportive evidence for the appropriate application and interpretation of serological tests in the diagnosis and management of patients with COVID-19.

## Supplementary Material

Supplemental Material
