# Supplementary figures and images for: Multicenter evaluation of two chemiluminescence and three lateral flow immunoassays for the diagnosis of COVID-19 and assessment of antibody dynamic responses to SARS-CoV-2 in Taiwan
Source: Emerg Microbes Infect. 2020 Oct 1;9(1):2157–68. doi: 10.1080/22221751.2020.1825016 (PMC7580576; doi:10.1080/22221751.2020.1825016)

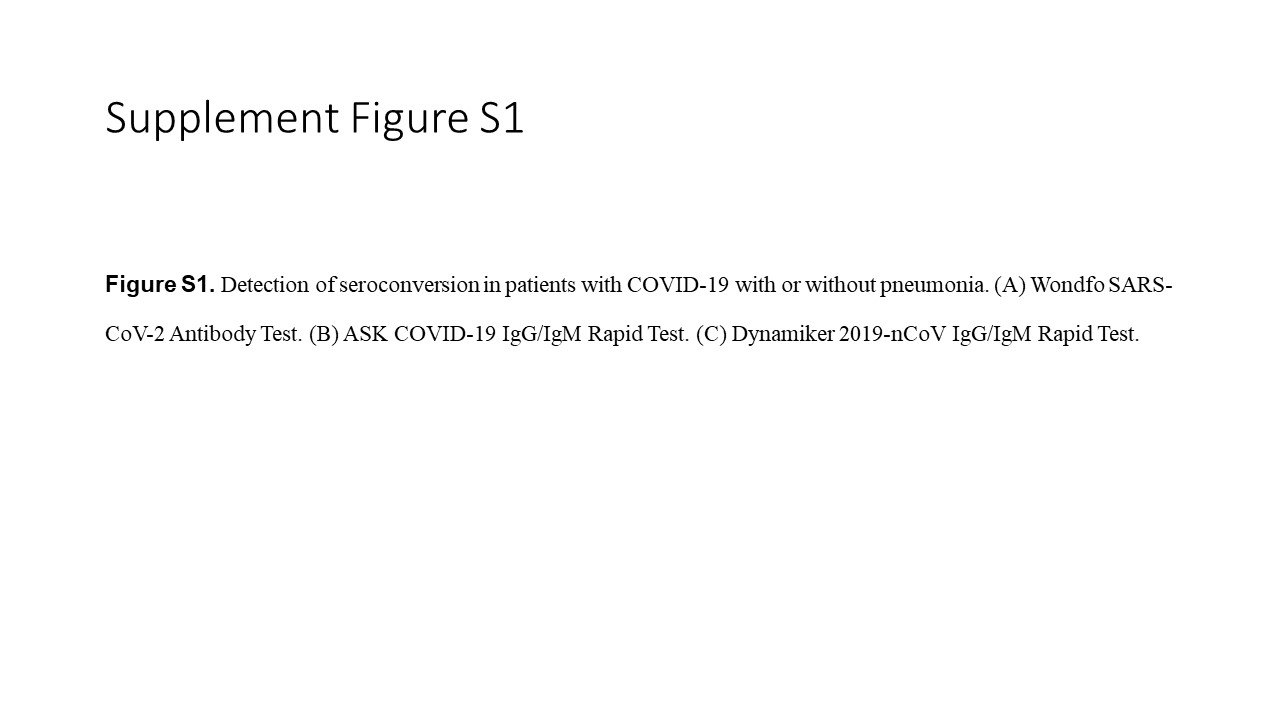

Supplement: Supplemental Material [file TEMI_A_1825016_SM3885.zip › Supplemental Figures/Fig._S1_Legend.JPG]

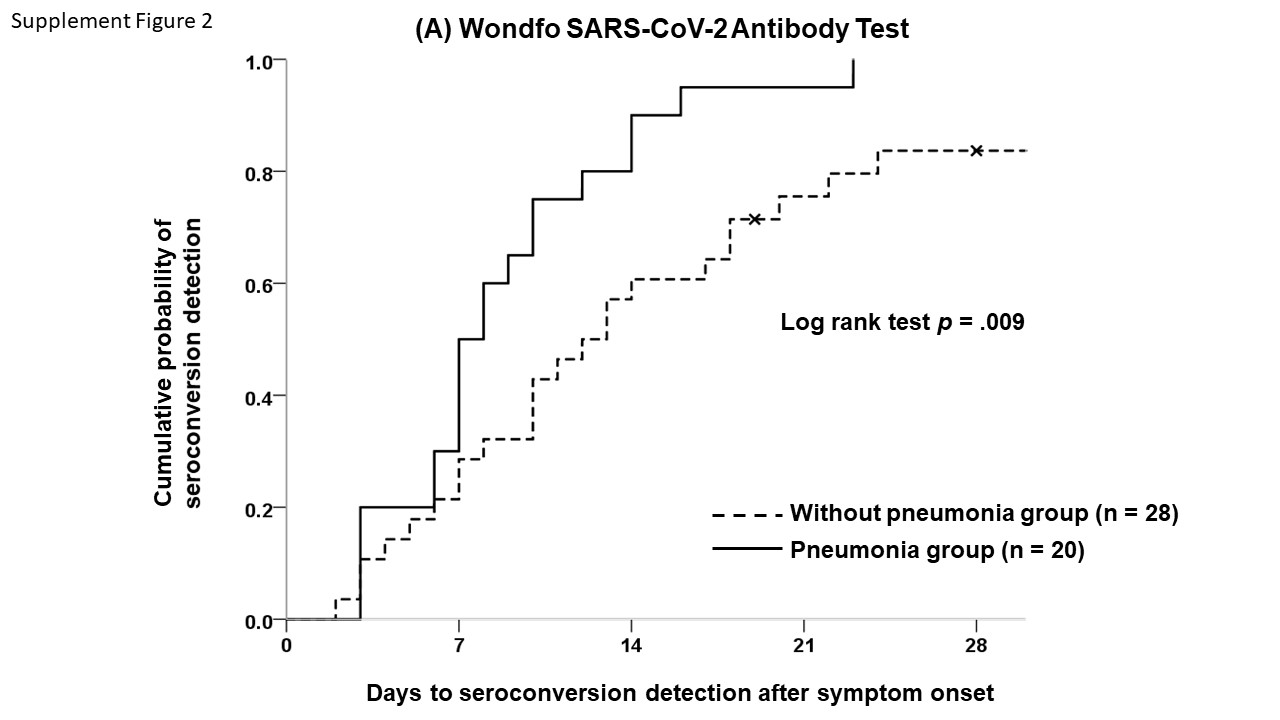

Supplement: Supplemental Material [file TEMI_A_1825016_SM3885.zip › Supplemental Figures/Fig._S1A.JPG]

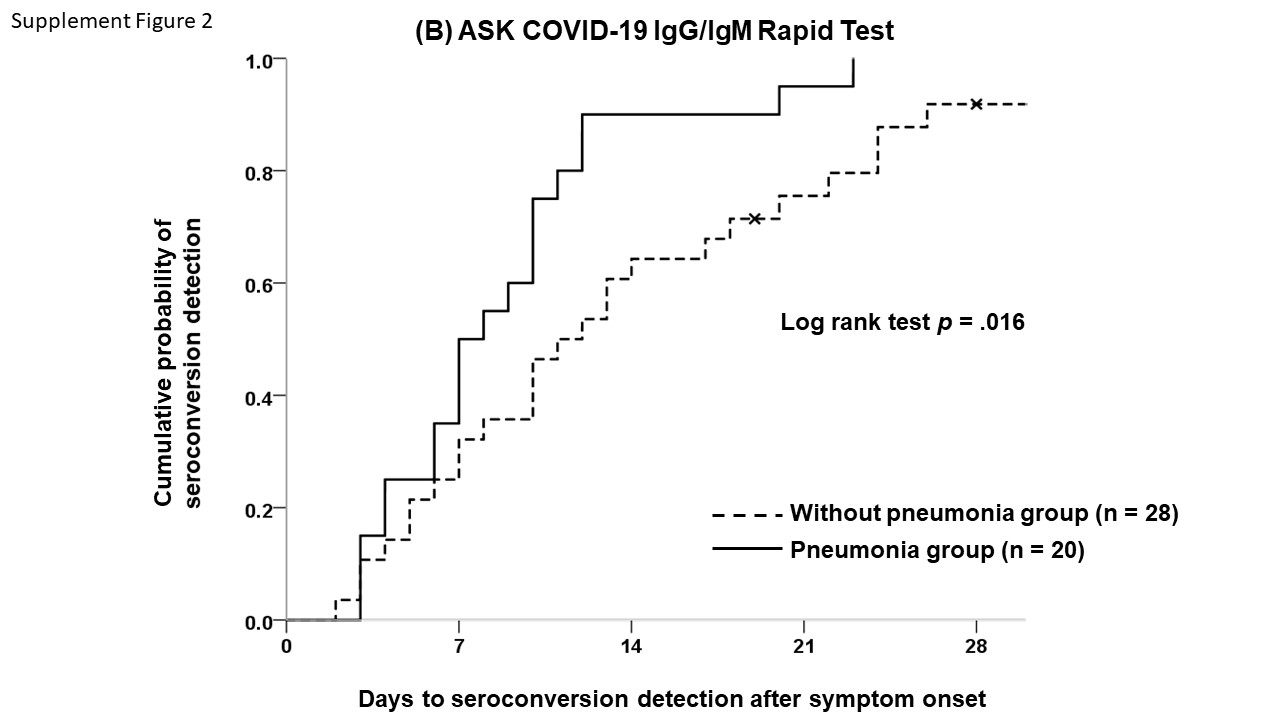

Supplement: Supplemental Material [file TEMI_A_1825016_SM3885.zip › Supplemental Figures/Fig._S1B.JPG]

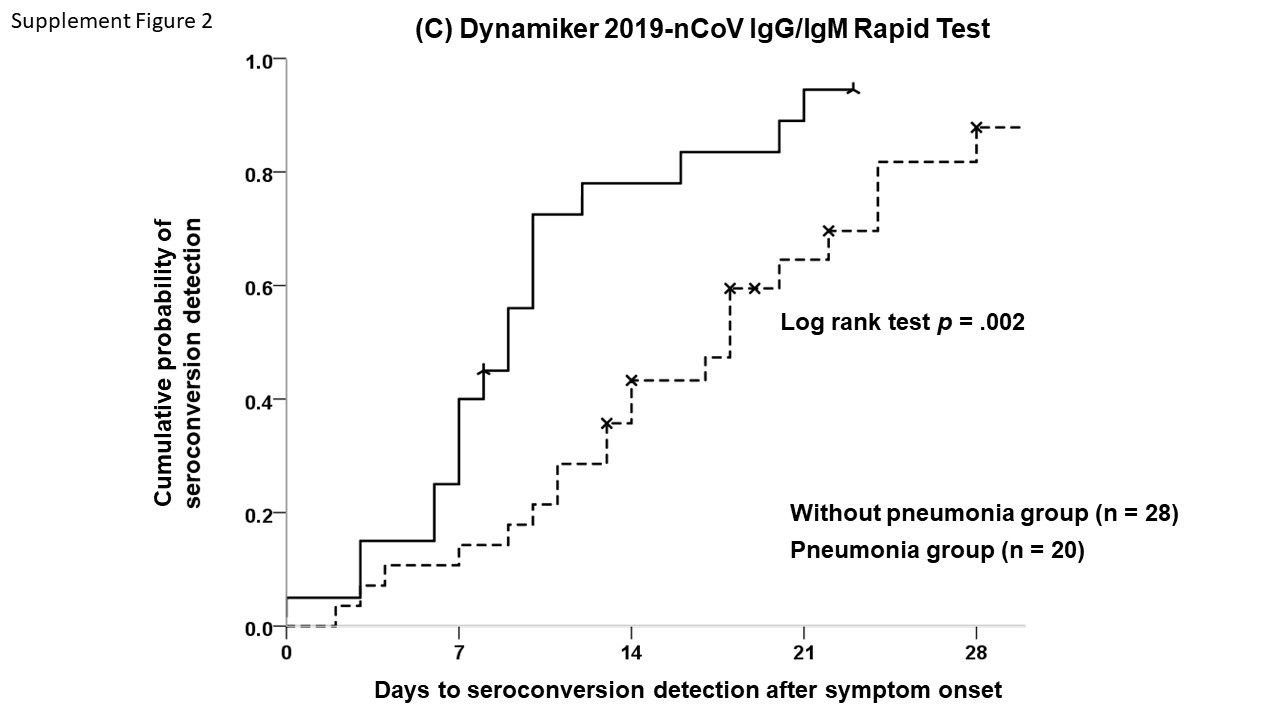

Supplement: Supplemental Material [file TEMI_A_1825016_SM3885.zip › Supplemental Figures/Fig._S1C.JPG]
